# Primary carnitine deficiency is a life‐long disease

**DOI:** 10.1002/jmd2.12319

**Published:** 2022-07-27

**Authors:** Loek L. Crefcoeur, Mireille C. Melles, Tobias A. Bruning, Rob Rodrigues Pereira, Janneke G. Langendonk

**Affiliations:** ^1^ Department of Metabolic Diseases Wilhelmina Children's Hospital, University Medical Center Utrecht Utrecht Netherlands; ^2^ Laboratory Genetic Metabolic Diseases Amsterdam UMC location Academic Medical Center, University of Amsterdam, Amsterdam Gastroenterology and Metabolism Amsterdam Netherlands; ^3^ Department of Cardiology Maasstad Hospital Rotterdam Netherlands; ^4^ Division of pediatrics Medical Center Kinderplein Rotterdam Netherlands; ^5^ Department of Internal Medicine, Center for Lysosomal and Metabolic Diseases Erasmus MC, University Medical Centre Rotterdam Rotterdam Netherlands

**Keywords:** cardiomyopathy, case report, heart failure, OCTN2 deficiency, primary carnitine deficiency

## Abstract

Primary carnitine deficiency is a rare autosomal recessive disease associated with acute hypoketotic hypoglycaemia, cardiomyopathy and sudden cardiac death. Effective treatment with carnitine supplementation is available. An 18 months old boy, who presented with cardiomyopathy was diagnosed with primary carnitine deficiency, and carnitine supplementation resulted in a full recovery. At age 13 years, he discontinued his medication and at 20 years, he discontinued clinical monitoring. Nine years later, age 29, he presented with heart failure and atrial fibrillation and was admitted to an intensive care unit, where he was treated with furosemide, enoximone and intravenous carnitine supplementation, this lead to improved cardiac function within 2 weeks, and with continued oral carnitine supplements, his left ventricular ejection fraction normalised. The last 8 years were uneventful and he continued to attend his regular follow‐up visits at a specialised metabolic outpatient clinic. We report recurrent reversible severe heart failure in a patient with primary carnitine deficiency; it was directly related to non‐compliance to carnitine supplementation (and monitoring). This case report emphasises first, the importance of continued monitoring of metabolic disease patients, second, the potential reversibility of cardiomyopathy in an adult patient, and third, the potential risks in the period of transition from the paediatric to adult care. This is an age where young adults desire to be healthy and ignore the need for ongoing medical treatment, even as simple as oral suppletion. Before they reach this age, adequate disease insight and self‐management of the disease should be promoted.

**Synopsis**

Continued monitoring is crucial to prevent recurrent complications in primary carnitine deficiency.

## 1 INTRODUCTION

Primary carnitine deficiency (OMIM entry #212140) is a rare autosomal recessive metabolic disease caused by pathogenic variations in the *SLC22A5* gene, encoding the Organic Cation Transporter Novel 2 (OCTN2) protein.^1^ This transporter protein maintains intracellular carnitine concentrations by transporting carnitine across the plasma membrane into cells, as well as reabsorbing carnitine in the proximal tubule of the kidney.^2^, ^3^ Defects in OCTN2 lead to significant decreases in intracellular carnitine availability, which may impair mitochondrial fatty acid oxidation.^4^, ^5^, ^6^ The disease can present early in life with acute hypoketotic hypoglycaemia, cardiomyopathy and sudden cardiac death.^7^ The condition is treatable with lifelong carnitine supplementation.

## 2 CASE REPORT

This report provides an update on a patient with primary carnitine deficiency that was reported earlier in two published case reports, 20 and 30 years ago.^8^, ^9^ A previously healthy 18 month old boy was admitted with lower respiratory tract infection (likely viral) followed by congestive heart failure. Cardiac ultrasound revealed a dilated left ventricle and poor contractility. Upon further investigation, serum free carnitine was extremely low 1.4 μmol/L (reference range 19–59 μmol/L) and urinary carnitine concentration was increased, which was indicative of primary carnitine deficiency. Due to advances in diagnostic procedures, the diagnosis primary carnitine deficiency was later genetically and functionally confirmed, identifying homozygous pathogenic variations in the *SLC22A5*‐gene (c.632A > G) and decreased carnitine transporter activity in cultured fibroblasts (activity level under 10% of mean control values).^9^ Therapy with diuretics, antibiotics and L‐carnitine (3TD 500 mg daily) resulted in normalised cardiac functions within 3 months after which only carnitine supplementation was continued. The following years were uneventful, with no change in his normal left ventricular ejection fraction (LVEF). From the age of 13 onwards, the patient became non‐compliant with his medication. Considering the patient decided to stop medication on a regular basis, his primary caretaker and parents repeatedly urged him to at least take carnitine supplements during illness. Despite not being on carnitine supplements, he participated in Ramadan for years without symptoms. Ramadan means fasting and abstinence from all food or drink, including water, from dawn to sunset (10–12 h per day), every year, during a period of 29–30 days. At the age of 20 years, he stopped visiting his metabolic specialist and only sporadically attended his cardiologist. At the age of 27, he withdrew himself from medical care completely. His last measured LVEF was 50% (normal is ≥50%), and serum free carnitine level was 4.0 μmol/L (reference range 22.3–54.8). A timeline of functional cardiac parameters and treatment data is presented in Table 1.

**TABLE 1:** Overview of cardiac parameters and carnitine status over time in the presented patient with primary carnitine deficiency

Age	Events	Prescribed carnitine suppletion dosage	Plasma free carnitine concentration (umol/L)	LVEF (%)	Shortening fraction (%)
			Normal value 22.3–54.8	Normal value ≥ 50%	Normal value ≥ 25%
18 months	Presented with severe cardiac failure	—	1		16–18
21 months	PCD diagnosed	3TD 0.5 g	NA		30
12 years		4TD 3.3 g	NA		31
13 years	Non‐compliance for medication	4TD 3.3 g	NA		
18 years		4TD 3.3 g	1	50	
19 years	Withdrew from metabolic specialist care	—	2		
27 years	Withdrew from all care	—	4	50	26
29 years	Admission ICU with severe cardiac failure	—	NA	Low^a^	
week 1		Intravenous: 6 g/24 h	35	30	
week 2		Intravenous: 6 g/24 h	49		
week 2	Discharge from ICU	4TD 1.5 g	NA		
31 years		4TD 2 g	30	42	21
33 years		4TD 3 g	26	54	
35 years		4TD 3 g	NA		33
37 years		4TD 3 g	14	55	

Abbreviations: ICU, Intensive care unit; LVEF, left ventricular ejection fraction; NA, unavailable; PCD, primary carnitine deficiency; TD, ‘times daily’.

a Very low, the ejection fraction was not quantified due to the poor image quality during severe cardiac failure, it will have been substantially lower than 30% since the next measurement with an LVEF of 30% was reported as a marked improvement.

At the age of 29 years the patient presented a second time with heart failure and atrial fibrillation, this was likely provoked by a viral respiratory tract infection. He was admitted to the intensive care unit. Ultrasound imaging revealed a dilated heart with severely decreased cardiac function and severe mitral valve insufficiency, LVEF could not be determined due to poor image quality. His free carnitine concentration was not measured upon admission. Treatment consisted of intravenous diuretics (furosemide), inotropes (enoximone) and L‐carnitine (6 gm bolus and then 6 gm daily continuously). After 2 weeks, he was discharged with a moderately improved LVEF of 30% (Table 1) and enrolled in a cardiac rehabilitation programme. In the following years he was followed up by his cardiologists and, with continued carnitine supplementation (varying from 4TD 1,5 ‐ 3 g daily), his LVEF improved to 57% with only moderate mitral valve insufficiency 5 years later.

At the age of 38 years, he was approached for participation in scientific research on primary carnitine deficiency, this resulted in a new appointment with an internist‐ metabolic consultant. His carnitine levels were again decreased: free carnitine 14 μmol/L, with carnitine supplementation of 4TD 3 g. His most recent cardiac evaluation showed a slightly dilated left ventricle, LVEF of 55%, and no arrhythmias. He was still considered unfit for work.

## 3 DISCUSSION

This case demonstrates that in diseases such as primary carnitine deficiency, where treatment is geared towards prevention, treatment adherence is a considerable challenge for both the patient and the caregiver, as the benefits from treatment are not immediately apparent.^10^, ^11^, ^12^, ^13^, ^14^, ^15^, ^16^ The consequence of discontinued carnitine supplementation was life threatening but only after years of discontinuation. Especially young adults are at risk of withdrawing from follow‐up, as they generally do not identify themselves as ‘sick’, having only a recollection of a symptom free life with good health. In these cases, treatment adherence may be improved with patient education aimed at improving disease insight.^11^, ^17^ We like to suggest to approach these patients as healthy and that our role as caregiver is to keep them healthy. Furthermore, patient engagement with the healthcare system may be improved with written information about the disease for other health care providers (e.g. the general practitioner), so they, too, can intervene and continue to urge restarting medication. In most expert centres there are special programs for safe and continued transition. Our patient indicated he discontinued his treatment before adolescence, as he believed there was no benefit from taking his medication and felt it was inconvenient. It is likely that, with continued treatment and follow‐up, the second cardiac decompensation could have been prevented.

This patients history illustrates the severe end of the phenotypic variation of primary carnitine deficiency. In an effort to prevent complications like these, primary carnitine deficiency was implemented in several newborn screening programs. After its introduction, asymptomatic individuals with primary carnitine deficiency are diagnosed in increasing numbers.^18^, ^19^, ^20^ It remains unclear if carnitine supplementation is beneficial for all individuals. Specifically in those that have had an uneventful childhood, being diagnosed by family counselling or as incidental finding following their child's newborn screening, the risk for severe disease outcome is unknown.^21^, ^22^ There have been reports of sudden cardiac death in seemingly asymptomatic adult PCD patients in the Faroese population, this will urge us to treat all diagnosed individuals.^23^, ^24^ The presented case, however, illustrates that continued carnitine supplementation seems essential for patients with initial symptomatic presentation, like heart failure. Even ‘healthy’ patients, without symptoms for decades while off treatment, can again develop severe complications.

Interestingly, at least for this patient, Ramadan related fasting did not provoke symptoms possibly related to PCD, despite very low‐free carnitine levels. This is surprising, as one would expect hypoketotic hypoglycaemia to occur during fasting. Fasting is an important provoking factor for disorders of mitochondrial long‐chain fatty acid oxidation and the carnitine shuttle.^4^, ^25^ In the presented case, both cardiac events were provoked by a viral upper respiratory tract infection. This is in line with other previously reported primary carnitine deficiency cases with severe cardiac decompensation, that were also preceded by (mild) upper respiratory tract infection.^26^, ^27^, ^28^, ^29^ Infections can be considered important provoking factors, and patients with low compliance should be reminded to take medication every day, but especially during (mild) infection.

In conclusion, patients with IEM that feel “healthy” following successful treatment during childhood years, may be prone to disregard their disease and important medication to maintain their health. Complications may occur after decades of non‐compliance. The role of caregivers to counsel young adults and to prevent non‐compliance, is of the utmost importance. Treatment cessation in primary carnitine deficiency can lead to severe heart failure, even decades later, and is unlike typical heart failure, completely reversible. In the event of cardiac decompensation all efforts should be made to bridge the gap to cardiac recovery.

## CONFLICT OF INTEREST

Loek Crefcoeur, Mireille Melles, Tobias Bruning, Rob Rodrigues Pereira and Janneke Langendonk declare no potential conflicts of interest.

## ETHICS STATEMENT

All procedures followed were in accordance with the ethical standards of the responsible committee on human experimentation (institutional and national) and with the Helsinki Declaration of 1975, as revised in 2000. Written informed consent for publication of this case report was obtained from the patient.

## Data Availability

Data sharing is not applicable to this article as no new data were created or analysed in this study.
